# Glycogen Phosphorylase B Is Regulated by miR101-3p and Promotes Hepatocellular Carcinoma Tumorigenesis

**DOI:** 10.3389/fcell.2020.566494

**Published:** 2020-11-25

**Authors:** Guangying Cui, Huifen Wang, Wenli Liu, Jiyuan Xing, Wengang Song, Zhaohai Zeng, Liwen Liu, Haiyu Wang, Xuemei Wang, Hong Luo, Xiaoyang Leng, Shen Shen

**Affiliations:** ^1^Department of Infectious Diseases, The First Affiliated Hospital of Zhengzhou University, Zhengzhou, China; ^2^Gene Hospital of Henan Province, Precision Medicine Center, The First Affiliated Hospital of Zhengzhou University, Zhengzhou, China; ^3^Clinical Laboratory Diagnostics, College of Medical Technology, Beihua University, Jilin, China; ^4^Department of Infectious Diseases, Guangshan County People’s Hospital, Xinyang, China

**Keywords:** glycogen phosphorylase B, HCC, tumorigenesis, prognosis, miR-101-3p

## Abstract

Glycogen metabolism plays a key role in tumorigenesis. High expression levels of glycogen phosphorylase B (PYGB) were reported in several cancers and might be served as a prognostic biomarker for cancer from precancerous lesions. Previous studies indicated the high expression of PYGB in hepatocellular carcinoma (HCC) tissues. However, the detailed roles of PYGB in HCC, as well as the regulatory mechanisms, are still unclear. In this study, we confirmed that PYGB was overexpressed in HCC tissues. PYGB overexpression was significantly associated with an aggressive tumor phenotype and poor prognosis of HCC patients. Functionally, PYGB knockdown suppressed HCC cell proliferation, migration and invasion *in vitro*, as well as tumorigenesis and metastasis *in vivo*. Bioinformatics analysis indicated that PYGB overexpression might enhance epithelial to mesenchymal transition (EMT) in HCC. Moreover, miR-101-3p was identified to post-transcriptionally inhibit the expression of PYGB via binding to 3′-UTR of PYGB. Overexpression of PYGB antagonized the regulatory effect of miR-101-3p on cell proliferation, migration and invasion in HCC cells. In summary, our results suggest that miR-101-3p/PYGB axis has an important role in HCC and PYGB could be served as a novel prognostic biomarker and therapeutic target for improving the prognosis of HCC patients.

## Introduction

Hepatocellular carcinoma (HCC) is the most common type of primary liver cancer and the third leading cause of cancer-related deaths worldwide (Bray et al., 2018). Unfortunately, its incidence rate continues to increase and the rising speed is faster than that of any other cancers both in men and women (Cronin et al., 2018; Siegel et al., 2019). Meanwhile, on account of the asymptomatic nature and late diagnosis of HCC patients, the clinical outcomes have not improved even with the development of therapeutic strategies, such as surgical resection, orthotropic liver transplantation and radio-frequency ablation (Farazi and DePinho, 2006; El-Serag and Rudolph, 2007; Ulahannan et al., 2014). Thus, it is urgent to find novel early diagnostic biomarkers and therapeutic strategies for improving the prognosis of HCC patients.

Glycogen phosphorylase, brain form (PYGB, also named GPBB), an enzyme encoded by the PYGB gene on chromosome 20, contributes to the regulation of carbohydrate metabolism by catalyzing the rate-determining first step from glycogen to glucose 1-phosphate (Gelinas et al., 1989; Pudil et al., 2010). Under stress conditions such as hypoxia, hypoglycemia, and ischemia, PYGB catalyzes glycogen degradation to supply emergency glucose, and then is released into the cytoplasm from the normal positioning-sarcoplasmic reticulum (SR) membrane (Pudil et al., 2010; Lillpopp et al., 2012). Interestingly, PYGB was firstly found to localize in nuclear of gastrointestinal cancer cells (Uno et al., 1998). Whereafter, up-regulated PYGB expression was reported in several cancers, such as colorectal cancer (Tashima et al., 2000), prostate cancer (Wang et al., 2018), non-small cell lung cancer (NSCLC) (Lee et al., 2006), ovarian cancer (Zhou et al., 2019). More importantly, PYGB overexpression was found during the conversion process from adenoma cells into carcinoma cells, which indicates that PYGB may be served as a prognostic biomarker for cancer from precancerous lesions (Uno et al., 1998; Tashima et al., 2000). In HCC, mouse monoclonal anti-Human Carcinoma antigen (HCAs) antibody specifically stained PYGB in HCC tissues, indicating that PYGB could be served as a potential biomarker for HCC diagnosis (Zhou et al., 2013). However, the detailed roles of PYGB in HCC, as well as the regulatory mechanisms, are still unclear.

MicroRNAs (miRNAs), a class of small non-coding RNAs with 21–25 nucleotides, function in RNA silencing and post-transcriptional regulation of gene expression by binding to the 3′-untranslated region (3′-UTR) of target mRNA (Bartel, 2004). Recently, mounting evidence documented a functional contribution of specific miRNAs in the development and progression of human cancers by regulating cancer-related cellular processes such as proliferation, migration, differentiation, apoptosis, and cell cycle progression (Garzon et al., 2010). There is also a series abnormal expression of miRNAs which involve in the regulation of HCC progression (Zheng et al., 2012). Thus, we focused on the miRNAs to explore the regulatory mechanisms of PYGB in HCC. miR-101, a down-regulated miRNA in HCC tissues, was associated with a poor prognosis of HCC patients and served as a tumor suppressor by targeting genes, such as EZH2, DUSP, Mcl-1, and so on (Su et al., 2009; Shen et al., 2014; Wei et al., 2015; Li C. Y. et al., 2017). However, the functions of miR-101 on PYGB in HCC have not been fully illuminated.

In this study, we confirmed that PYGB was highly expressed in HCC tissues and associated with poor prognosis in HCC patients. Knockdown of PYGB inhibited HCC cell proliferation, migration, and invasion *in vitro* and suppressed HCC tumorigenesis in xenograft tumor model. Mechanistically, we demonstrated that PYGB was post-transcriptionally regulated by miR-101-3p. Collectively, our results suggest that high expression of PYGB may be served as a novel prognostic biomarker and therapeutic target for improving the prognosis of HCC patients.

## Materials and Methods

### Patients and Specimens

The diagnosis of HCC patients in this study was based on histopathology and/or CT/MRI. 324 paired tumor and adjacent non-tumor samples (follow-up data collected until March 2018) were obtained from HCC patients who were admitted to the First Affiliated Hospital of Zhengzhou University during 2009–2012 (Zhengzhou, China), named as ZZU HCC cohort. The clinicopathologic features of these patients were shown in Table 1. Written informed consent was obtained from each patient. The study was performed in accordance with the Helsinki Declaration and Rules of Good Clinical Practice and approval by the First Affiliated Hospital of Zhengzhou University.

**TABLE 1:** Correlation of clinico-pathological features with PYGB expression in ZZU HCC cohort.

		**PYGB expression**		
**Variables**	**Clinicopathological**	**Low expression**	**High expression**	***P-value***
	**features**	**(*n* = 47)**	**(*n* = 194)**	
Age (years)	≤50	58 (39.4)	68 (35.1)	0.404
	>50	89 (60.6)	126 (64.9)	
Gender	Male	110 (74.8)	145 (79.8)	0.265
	Female	37 (25.1)	36 (20.1)	
Pathogenesis	Virus	119 (78.2)	136 (71.9)	0.181
	Others	33 (21.7)	53 (28.1)	
Cirrhosis	Absent	132 (89.7)	183 (94.3)	0.118
	Present	15 (10.3)	11 (5.7)	
AFP	20	68 (46.2)	98 (50.5)	0.436
	>20	79 (53.7)	96 (49.5)	
Child-Pugh	A/B	90 (61.2)	115 (59.2)	0.716
	C	57 (38.7)	79 (40.7)	
Vascular invasion	Absent	128 (87.1)	141 (72.6)	**0.001**
	Present	19 (12.9)	53 (27.3)	
TNM stage	Stage I and II	122 (82.9)	128 (72.6)	**0.024**
	Stage III and IV	25 (17.1)	53 (27.3)	
Tumor size (cm)	5	81 (55.1)	91 (46.9)	0.133
	>5	66 (44.9)	103 (53.1)	
Tumor multiplicity	Single	68 (46.2)	91 (46.9)	0.905
	Multiple	79 (53.8)	103 (53.1)	
Survival state	Live	108 (73.4)	122 (62.9)	**0.039**
	Dead	39 (26.6)	72 (37.1)	

*Bold values indicate statistical significance, *P* < 0.05*.

### Tissue Microarrays (TMA) and Immunohistochemistry (IHC)

Tissue microarrays were constructed as previously described (Xie et al., 2017). Briefly, specimens were stained with hematoxylin and eosin (H&E). With the help of two pathologists, two biopsies of 1 mm in diameter were taken and transferred to the defined array positions to perform HCC TMAs.

Immunohistochemistry was performed using a two-step protocol as described in our previous study (Chen et al., 2019). Briefly, 4 μm-thick serial sections from HCC TMAs were deparaffinized and rehydrated. Second, referring to the manufacturer’s instructions, antigen retrieval was performed with Target Retrieval Solution (Dako, CA, United States). Next, tissue sections were incubated with primary antibody (PYGB, 1:200 dilution, Proteintech; Ki-67, 1:500 dilution, Signalway Antibody) and secondary antibody. To visualize staining, sections were incubated with DAB (3,3diaminobenzidine) and terminated in phosphate-buffered saline (PBS), and counterstained with hematoxylin QS (Vector Laboratories). Staining images were recorded. Finally, IHC scores were evaluated by two pathologists in a blinded manner and the PYGB staining was scored on a scale of 1–5 based on intensity and area as shown in Figure 2B. For further analysis of the relationship between PYGB expression and the clinicopathologic features in HCC patients, HCC patients were divided into high PYGB group (scores 3 and 4) and low PYGB group (scores 1 and 2).

### TCGA/GEO Datasets Acquisition and Process

The expression levels of PYGB mRNA in HCC were downloaded directly from Gene Expression Omnibus (GEO)^1^ datasets. A series of tumor datasets were also downloaded directly from the open access tiers of The Cancer Genome Atlas (TCGA^2^) (hereinafter referred to as the TCGA cohort), including TCGA HCC cohort with at least 10 years of follow-up (including 367 HCC patients with complete PYGB mRNA expression, miR-101-3p expression data and corresponding clinical data).

The process of dataset was performed as previously described (da Huang et al., 2009; Li G. et al., 2017). GO and Kyoto encyclopedia of genes and genomes (KEGG) pathway enrichment analysis were performed using the TCGA HCC cohort to list the genes correlated with PYGB Meanwhile, Gene set enrichment analysis (GSEA) was performed to collect PYGB-correlated gene set. A nominal *P*-value of *P* < 0.05 was considered as the significance enrichment.

### Cell Culture and Transfections

Human HCC cell lines (HepG2, Sk-Hep-3b, MHCC97-H and SMMC-7721), hepatocyte cell lines (Chang liver and L02) were purchased from the Cell Bank of the Chinese Academy of Sciences (Shanghai, China) and mice HCC cell line Hepa 1-6 was obtained from the First Affiliated Hospital of Zhejiang University. All cell lines were maintained in Dulbecco’s modified Eagle’s medium (DMEM) supplemented with 10% fetal bovine serum (FBS) (Gibco, NY, United States) and 100 U/ml penicillin/streptomycin (Corning, NY, United States) in a CO2 incubator at 37°C.

For transfection, HCC cells (1 × 10^6^ per well) were seeded into a six-well plate. ShRNAs targeting PYGB (Sh-PYGB-1, Sh-PYGB-2, and Sh-PYGB-2), a scramble RNA (Lenti-shCtrl), pcDNA-PYGB, miR-101-3p mimics, mimics NC, miR-101-3p inhibitor, inhibitor NC, wt-PYGB and/or mut-PYGB reporter vector (synthesized by GenePharma) were transfected into cells using Lipofectamine 2000 (Invitrogen, CA, United States) according to the manufacturer’s protocol.

### Western Blotting

Western blotting was performed as described in our previous study (Chen et al., 2019). Briefly, cells were lysed and samples were prepared using RIPA protein extraction reagent (Beyotime, Shanghai, China) and a protease inhibitor cocktail (Roche, IN, United States) according to the manufacturer’s protocol. Next, samples with equal amounts of protein were electrophoresed on 10% SDS-PAGE gels and then transferred onto PVDF membranes (Millipore, MA, United States). Membranes were blocked in 5% fat-free milk in TBST buffer, then incubated with primary antibodies (GAPDH antibody, 1:5000 dilution, Proteintech; anti-rabbit PYGB antibody, Ki-67 antibody, *N*-cadherin, *E*-cadherin, β-catenin, Snail, Slug, and Twist, 1:1000 dilution; Signalway Antibody, TX, United States) at 4°C overnight. Finally, the second antibody (Horseradish peroxidase-conjugated goat anti-rabbit IgG antibody 1:5000 dilution, Beyotime) was used and the signals were visualized using an Odyssey detection system (Li-COR biosciences, NE, United States).

### Luciferase Reporter Assay

Hepatocellular carcinoma cells were transfected with reporter vector containing wild-type or mutated 3′-UTR of PYGB, together with miR-101-3p mimic or mimics negative control. The relative luciferase activity of HCC cells was normalized to Renilla luciferase activity 48 h after transfection using the Dual-Glo luciferase reporter assay kit (Promega, Madison, WI, United States).

### RNA Extraction, Reverse Transcription, and Quantitative Real-Time PCR

The detailed procedure was performed as our previous described (Chen et al., 2019). In brief, total RNA was extracted from tissues or cells using Trizol Reagent (Invitrogen, Carlsbad, CA, United States). Complementary DNA was reverse transcribed from RNA using PrimeScript^TM^ RT Master Mix (TAKARA, Dalian. China). Quantitative real-time PCR was performed using SYBR^®^ Fast qPCR Mix (TAKARA) on 7900HT fast Real-time PCR system (Applied Biosystems, Foster City, CA, United States). The relative expression of gene was normalized to GAPDH or U6 using 2^–ΔΔ*Ct*^ methods, respectively.

### Cell Growth Assays

For cell proliferation assays, HCC cells (5 × 10^3^ cells per well) were seeded into triplicate wells of 96-well plates. Cell counting kit-8 (CCK-8) (Dojindo, Kyushu, Japan) was used to determine cell numbers at 24, 48, 72, and 96 h, according to the manufacturer’s protocol. The absorbance was measured at 450 nm using an ELISA reader (TECAN, Mannedorf, Switzerland). Meanwhile, DNA synthesis rate was assayed by using a 5-ethynyl-20-deoxyuridine (EdU) assay kit (Ribobio, Guangzhou, China), and images were taken with a microscope (Olympus, Tokyo, Japan) at 100 × magnification. Cell proliferation activity was evaluated by the ratio of EdU-stained cells (with red fluorescence) to Hoechst-stained cells (with blue fluorescence). For colony formation assay, HCC cells (1 × 10^3^ cells per well) were seeded into 6-well plates. After 14 days, cells were fixed by 30% formaldehyde for 15 min and stained with 0.1% crystal violet. The number of colonies (containing more than 50 cells) was determined under an optical microscope (Olympus, Tokyo, Japan).

### Cell Migration and Invasion Assays

Cell migration was assessed using a wound-healing assay. Briefly, cells were plated into a 6-well plate and artificial scratches were created using a sterile 200 μl pipette tip when 90% confluence was reached. Then, cell migration was monitored at 0 and 48 h using an Olympus 1X71 camera system.

Cell invasive ability was evaluated using a transwell assay. Cells (5 × 10^4^ cells per well) were plated into the upper chamber of a transwell plate (BD Biosciences, San Jose, CA, United States) coated with Matrigel. DMEM supplemented with 20% FBS was used as a chemoattractant in lower chamber. Following 36 h incubation, invading cells were fixed in 4% paraformaldehyde for 20 min, stained with 0.5% crystal violet and then counted under a microscope.

### Animal Tumor Models

Animal experiments were approved and performed according to Institutional Ethical Guidelines for Animal Experiments in Zhengzhou University.

#### Nude Tumor-Bearing Mice Model

Briefly, 8-weeks-old male BALB/c nude mice (purchased from Vital River Technology Co., Ltd, China, product code: 403) were maintained in the animal facility of the First Affiliated Hospital of Zhengzhou University in a controlled environment. Approximately 3 × 10^6^ MHCC97-H cells stably transfected with ShPYGB-2 (ShPYGB group) or ShCtrl (ShCtrl group) were subcutaneously injected into BALB/c nude mice. The tumor volume was measured by the formula as previously (Bao et al., 2018): Volume = (width)^2^ × length/2; 10 min after intraperitoneal injection with 4.0 mg of luciferin (Gold Biotechnology, Inc., St. Louis, MO, United States) in 50 μl of saline, tumor images were recorded with an IVIS@ Lumina II system (Caliper Life Sciences, Hopkinton, MA, United States). Tumors were surgically removed and weighed after 5 weeks.

#### The Orthotopic Mice Model

The orthotopic HCC mouse model was established as previously described (Wu et al., 2019). Briefly, 5 × 10^5^ Hepa 1-6 cells stably transfected with ShPYGB-2 or ShCtrl suspended in a mixture of 12.5 μL PBS and 12.5 μL Basement Membrane Matrix (Corning, Matrigel) was injected into left liver lobe of 8-weeks-old male C57BL/6 mouse. After 2 weeks, the mice were sacrificed and the ratio of liver weight/tumor volume was analyzed for tumor volume.

#### Pulmonary Metastasis Model

The mouse model of pulmonary metastasis via subcutaneous injection was established as previously described (Li et al., 2003; Peng et al., 2013). Briefly, 1 × 10^7^ MHCC97-H cells stably transfected with ShPYGB-2 or ShCtrl were subcutaneously into nude mice. After 6 weeks, the mice were sacrificed and the lung histopathology was analyzed for metastasis assay.

### Statistical Analysis

All experiments were performed in triplicate. SPSS (version 23.0, SPSS Inc.) or GraphPad Prism software (version 7.0, United States) was used for statistical analysis. Chi-square tests, Pearson’ analysis, log-rank tests, univariate and multivariate Cox regression analysis, Student’s *t*-test and the Mann–Whitney *U* test were used for statistical analysis where necessary. *P*(two-sided) < 0.05 was considered statistical significant.

## Results

### High Expression of PYGB Is Associated With Poor Prognosis of HCC Patients in TCGA Cohort and GEO Cohort

**FIGURE 1:**
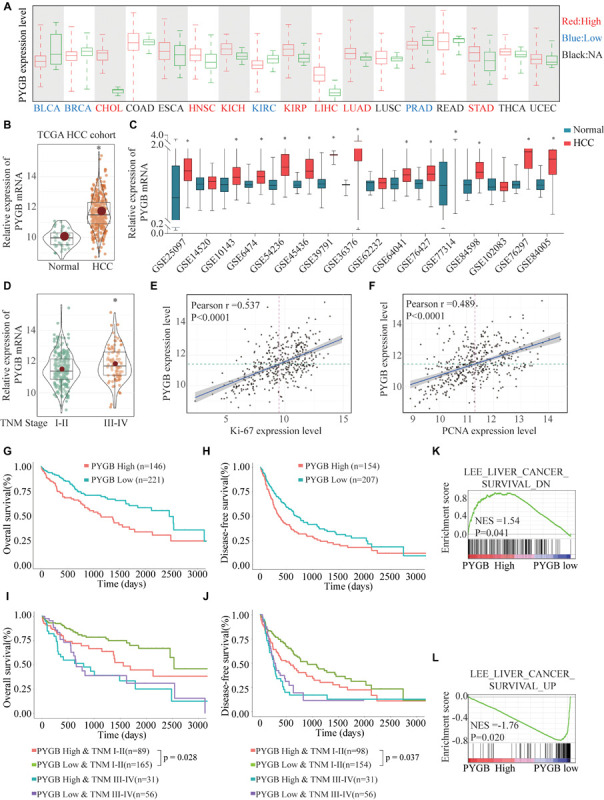
The PYGB mRNA expression and its relationship with clinico-pathological features of HCC patients were analyzed using TCGA cohort and GEO cohort. **(A)** The mRNA expression of PYGB in a series of cancer tissues and the corresponding normal tissues was analyzed in TCGA cohort. BLCA, bladder urothelial carcinoma; BRCA, breast cancer; CHOL, cholangiocarcinoma; COAD, colon cancer; ESCA, esophagus cancer; HNSC, head and neck squamous cell carcinoma; KIRC, renal cancer; KIRC, kidney renal clear cell carcinoma; KIRP, kidney renal papillary cell carcinoma; LIHC, liver cancer; LUAD, lung cancer; LUSC, lung squamous cell carcinoma; PRAD, prostate adenocarcinoma; READ, rectal cancer; STAD, stomach cancer; THCA, thyroid cancer; UCEC, uterine corpus endometrial carcinoma. **(B)** The mRNA expression of PYGB in HCC tissues and normal liver tissues was analyzed in TCGA HCC cohort. **(C)** The mRNA expression of PYGB was analyzed in HCC tissues and normal liver tissues in 16 GEO HCC datasets. **(D)** The mRNA expression of PYGB was analyzed in patients with different TNM stages in TCGA HCC cohort. **(E,F)** The relationships between PYGB mRNA expression and the mRNA expression levels of Ki-67 and PCNA were assessed by Pearson analysis. **(G,H)** Kaplan–Meier analysis the overall survival (OS) and disease-free survival (DFS) in HCC patients with high or low PYGB expression in TCGA HCC cohort. **(I,J)** Hierarchical analyses of OS and DFS in HCC patients with high or low expression of PYGB based on TNM stages. **(K,L)** Gene Set Enrichment Analysis (GSEA) of survival up- and down-associated signature genes in HCC patients with high or low PYGB expression in TCGA HCC cohort. *p < 0.05.

To investigate the role of PYGB in tumor progression, we first analyzed PYGB expression in different malignancies in TCGA cohort. PYGB expression was dysregulated in a series of cancer tissues compared with the corresponding normal tissue (Figure 1A). The expression of PYGB was significantly higher in HCC tissues than that in normal tissues (Figure 1B). High PYGB expression was further confirmed in 13 of 16 GEO HCC datasets (Figure 1C). In addition, the expression of PYGB was enhanced in HCC patients with advanced TNM stage (TNM III-IV *vs.* TNM I-II) (Figure 1D). Pearson correlation analysis revealed that high PYGB expression was positively correlated with the expression levels of proliferation biomarkers (Ki-67 and PCNA) (Figures 1E,F). Importantly, patients with high PYGB expression had worse overall survival (OS) and disease-free survival (DFS) than that in patients with low PYGB expression by Kaplan–Meier analysis (Figures 1G,H). Hierarchical analyses based on TNM stages were performed and patients with high PYGB expression had markedly poorer OS and DFS was observed in HCC TNM I-II (Figures 1I,J). Furthermore, in order to explore the clinical significance of PYGB in HCC prognosis, GSEA was performed based on mRNA expression data from the TCGA HCC cohort, which indicated that high PYGB expression was closely correlated with survival down-associated gene signatures (Figures 1K,L). These results suggest that PYGB mRNA is highly expressed and associated with poor prognosis of HCC patients.

### High PYGB Protein Expression Is Positively Correlated With Poor Prognosis of HCC Patients in ZZU HCC Cohort

**FIGURE 2:**
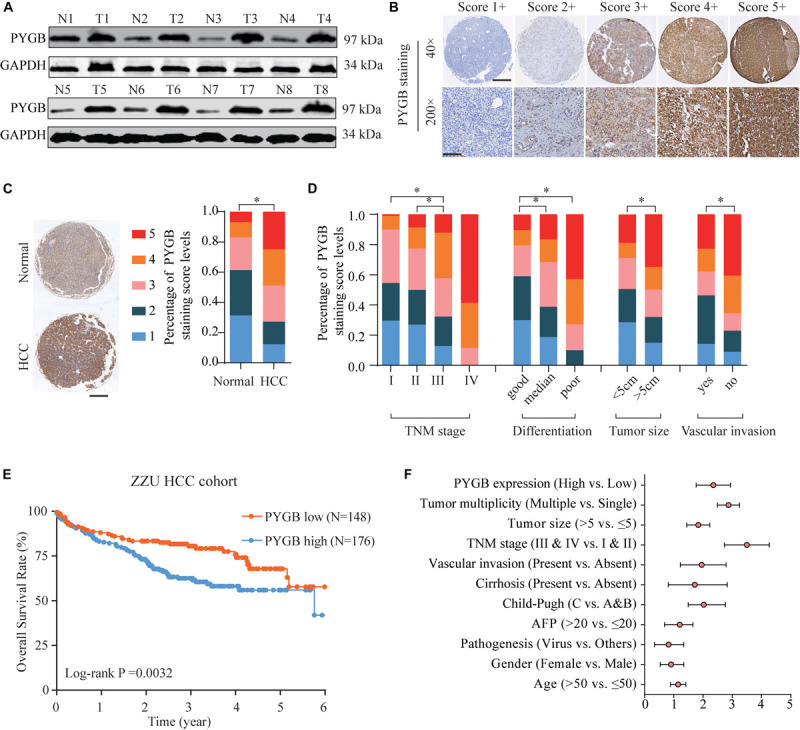
High PYGB protein expression is positively correlated with poor prognosis of HCC patients in ZZU HCC cohort. **(A)** PYGB protein expression was analyzed in paired tumor and adjacent non-tumor samples from 8 HCC patients by western blot. **(B)** PYGB protein expression was examined by IHC staining in ZZU HCC cohort and scored on a scale of 1–5 according to the staining intensity and area. **(C)** PYGB protein expression was analyzed in HCC tissues and adjacent non-tumor liver tissues based on the PYGB IHC staining score. **(D)** PYGB expressions were analyzed in HCC patients with different TNM stages, poor differentiation, tumor size and vascular invasion based on the PYGB IHC staining score. **(E)** The overall survival rates in HCC patients with high or low PYGB expression were analyzed by Kaplan-Meier analysis in ZZU HCC cohort. **(F)** Univariate and Multivariate Cox regression analysis of the clinico-pathological features and PYGB expression in ZZU HCC cohort. *p < 0.05.

Based on the heterogeneity between mRNA expression and protein expression of genes, we further examined the protein expression of PYGB in HCC tissues using ZZU HCC cohort containing 324 paired HCC tumor and adjacent non-tumor samples. Higher levels of PYGB protein expression were observed in HCC tumor tissues compared with that in adjacent non-tumor samples (Figure 2A). PYGB protein expression was also detected by immunohistochemical staining using ZZU HCC cohort tissue microarray (TMA) and scored on a scale of 1–5, according to the staining intensity and area of staining (Figure 2B). Compared with that in adjacent non-tumor liver tissues, PYGB protein was highly expressed in HCC tissues (Figure 2C). Moreover, high PYGB expression was observed in HCC patients with advanced TNM stages, poor differentiation, large tumor size and vascular invasion (Figure 2D). Importantly, Kaplan–Meier analysis showed that patients with high PYGB expression had poorer OS than patients with low PYGB expression (Figure 2E). In addition, univariate and multivariate Cox regression analyses revealed that PYGB expression, as well as TNM stages and vascular invasion, was an independent risk factor for HCC prognosis (Figure 2F and Table 2). These results confirm that PYGB is highly expressed in HCC tumor tissues and high PYGB expression indicates a poor prognosis of HCC patients.

**TABLE 2:** Correlation of clinico-pathological features with PYGB expression in ZZU HCC cohort.

	**Univariate analysis**			**Multivariate analysis**		
	**HR**	**95% CI**	***P*-value**	**HR**	**95% CI**	***P*-value**
Age (>50 vs. ≤50)	1.143	0.884–1.413	0.863			
Gender (Female vs. ≤Male)	0.846	0.534–1.342	0.479			
Pathogenesis (Virus vs. ≤Others)	0.784	0.341–1.341	0.243			
AFP (>20 vs. ≤20)	1.270	0.684–1.654	0.347			
Child-Pugh (C vs. A&B)	1.814	1.491–2.765	**0.029**	1.644	1.121–1.936	0.248
Cirrhosis (Present vs. Absent)	1.515	0.812–2.825	0.192			
Vascular invasion (Present vs. Absent)	1.848	1.225–2.793	**0.003**	3.973	1.942–5.419	**0.001**
TNM stage (III and IV vs. I and II)	3.333	2.262–4.902	**0.000**	3.473	2.479–5.349	**0.008**
Tumor size (>5 vs. ≤5)	1.751	1.198–2.564	**0.004**	2.045	1.221–2.732	0.078
Tumor multiplicity (Multiple vs. Single)	2.964	2.176–3.47	**0.019**	1.975	1.428–2.668	0.056
PYGB expression (High vs. Low)	2.091	1.492–3.473	**0.027**	2.455	1.715–3.677	**0.015**

*Bold values indicate statistical significance, *P* < 0.05.*

### Knockdown of PYGB Expression Inhibits Cell Growth, Invasion and Migration of HCC Cells *in vitro*

**FIGURE 3:**
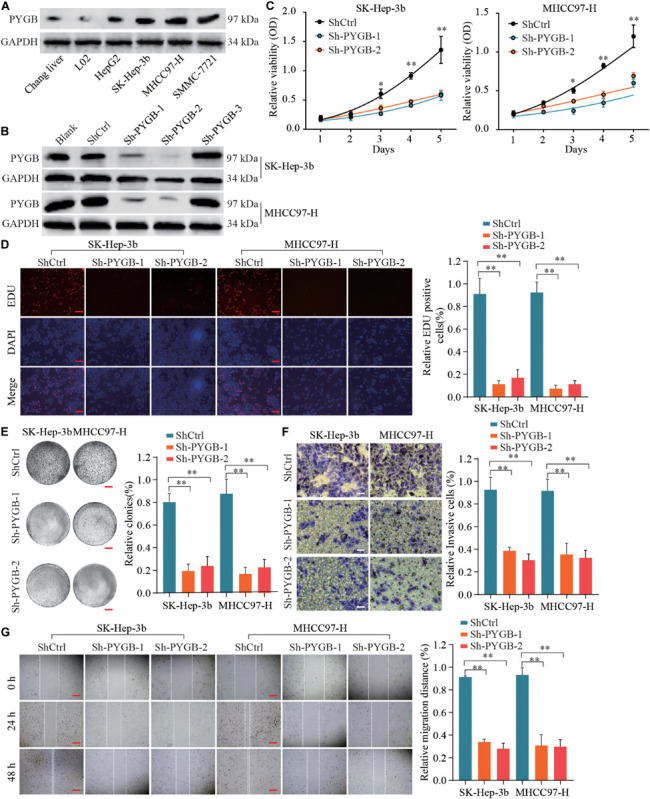
Knockdown of PYGB expression inhibits cell growth, invasion and migration of HCC cells *in vitro*. **(A)** PYGB protein expression in HCC cell lines (HepG2, SK-Hep-3b, MHCC97-H and SMMC-7721) and hepatocyte cell lines (Chang liver and L02) was examined by western blot assay. **(B)** Two HCC cell lines (SK-Hep-3b and MHCC97-H) were transfected with shRNA lentiviral vector (Sh-PYGB-1/2/3) or control (shCtrl) and the PYGB knockdown efficiency was analyzed by western blot. SK-Hep-3b or MHCC97-H cells were transfected with shCtrl, sh-PYGB-1 or sh-PYGB-2. Cell growth was evaluated by CCK-8 assay **(C)**, EdU assay **(D)** and colony formation assay **(E)**. HCC cell invasion and migration was analyzed by Transwell assay **(F)** and Wound-healing assay **(G)**. *p < 0.05, **p < 0.01.

To characterize the function of PYGB in HCC, the protein expression levels of PYGB in HCC cell lines (HepG2, Sk-Hep-3b, MHCC97-H and SMMC-771) and hepatocyte cell lines (Chang liver and L02) were examined. High PYGB expression was observed in HCC cell lines compared with that in hepatocyte cell lines (Figure 3A). Then, shRNA vectors targeting PYGB were transfected into two HCC cell lines (Sk-Hep-3b and MHCC97-H) and the PYGB knockdown efficiency was determined by western blot (Figure 3B). Two efficient shRNAs (sh-PYGB-1 and sh-PYGB-2) were used to further assess the function of PYGB. CCK-8 assay and EdU assay were performed. The results revealed that knockdown of PYGB expression suppressed the cell growth and DNA synthesis of HCC cells (Figures 3C,D). Meanwhile, colony formation assay also confirmed that downregulation of PYGB expression inhibited cell growth (Figure 3E). Furthermore, knockdown of PYGB inhibited cell invasion as demonstrated by transwell assay (Figure 3F). In addition, the capability of cell migration was dampened in HCC cells transfected with sh-PYGB vectors (Figure 3G). These data suggest that PYGB plays a critical role in cell growth, invasion and migration of HCC cells.

### Knockdown of PYGB Inhibits Tumorigenesis and Metastasis of HCC *in vivo*

**FIGURE 4:**
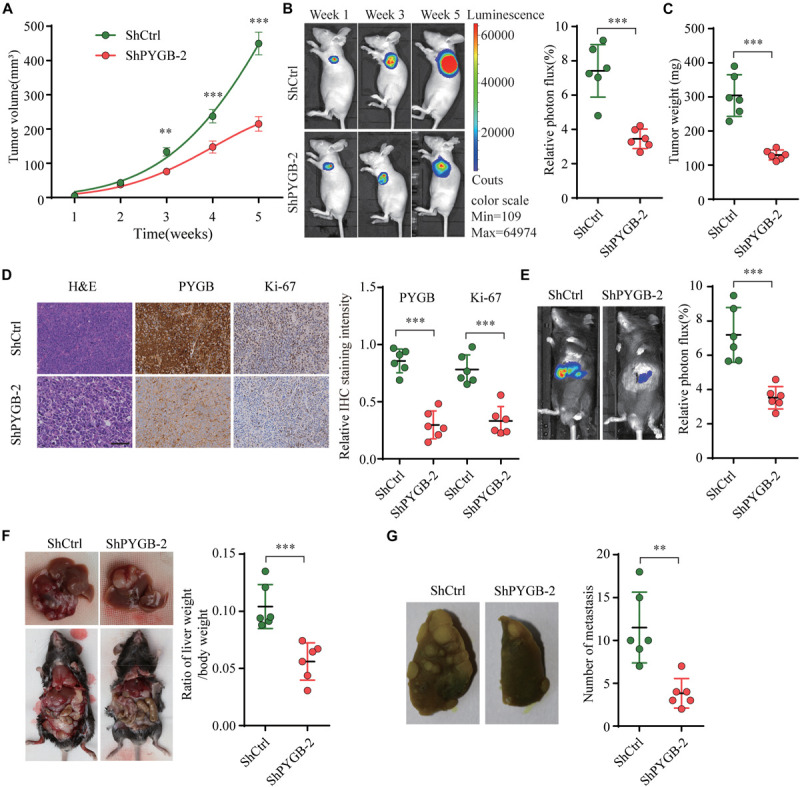
Knockdown of PYGB inhibits tumorigenesis and metastasis of HCC *in vivo*. MHCC97-H cells stably transfected with shPYGB or shCtrl were subcutaneously injected into BALB/c nude mice. **(A)** Tumor growth was monitored and tumor volume was measured at indicated time points. **(B)** Tumor image of luciferin photon flux was recorded at week 1, 3, and 5 after cell implantation. **(C)** Tumor weight was analyzed in ShPYGB group and ShCtrl group. **(D)** PYGB and Ki-67 expression in tumor tissues from ShPYGB group and ShCtrl group were analyzed by immunohistochemical staining. Hepa 1-6 cells stably transfected with shPYGB or shCtrl were injected into left liver lobe of C57BL/6 mice to establish the *orthotopic* tumor model. **(E,F)** Tumor size and tumor weight were analyzed by relative photon flux and the ratio of liver weight/body weight in ShPYGB group and ShCtrl group. **(G)** MHCC97-H cells stably transfected with shPYGB or shCtrl were subcutaneously injected into nude mice. Six weeks later, mice were sacrificed and the pulmonary metastasis was analyzed by lung histopahtology. **p < 0.01, ***p < 0.001.

To further investigate the function of PYGB in HCC progression *in vivo*, three tumor-bearing mouse models were employed. In nude tumor-bearing xenograft mouse model, we found that knockdown of PYGB markedly suppressed tumor growth. Tumor size and tumor weight were dramatically reduced in ShPYGB group compared with that in ShCtrl group (Figures 4A–C). Consistently, IHC staining showed that the expression of PYGB and Ki67 in tumor tissues from ShPYGB group was much lower in comparison with that in ShCtrl group (Figure 4D). Furthermore, The orthotopic tumor model demonstrated that knockdown of PYGB inhibited tumorigenesis, with smaller tumor size and lower tumor weight in shPYGB group (Figures 4E,F). Hepa 1–6 cells stably transfected with shPYGB revealed a less potent metastasis ability, with fewer tumor nodes compared with that in mice implanted with Hepa 1–6 cells stably transfected with ShCtrl (Figure 4G). Taken together, these results indicate that knockdown of PYGB suppresses HCC tumorigenesis and pulmonary metastasis *in vivo*.

### Knockdown of PYGB Inhibits Epithelial to Mesenchymal Transition (EMT) in HCC

**FIGURE 5:**
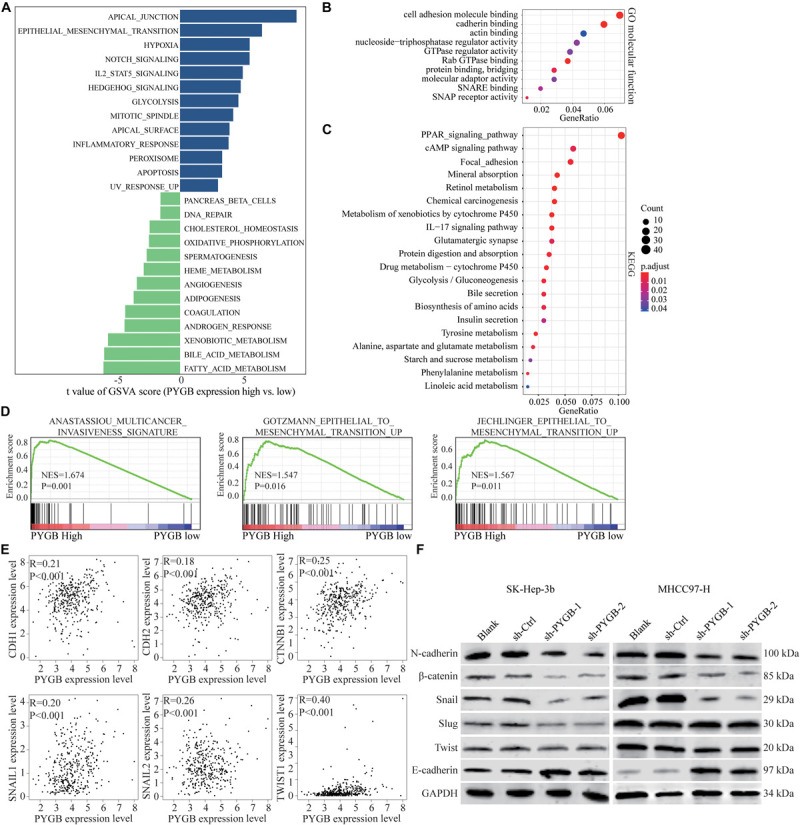
Knockdown of PYGB inhibits epithelial to mesenchymal transition (EMT) in HCC. Bioinformatic analysis was performed using TCGA HCC datasets. **(A)** Differential expression of genes in HCC patients with high or low PYGB expression was analyzed by GSVA. **(B,C)** Gene functions were analyzed by GO and KEGG pathway enrichment analyses. **(D)** GSEA analysis the correlation between PYGB expression and invasiveness- and epithelial to mesenchymal transition (EMT)-associated gene signature. **(E)** Pearson analysis of the relationships between PYGB and the mRNA expressions of the genes including *E*-cadherin (CDH1), *N*-cadherin (CDH2), β-catenin (CTNNB1), Snail (SNAIL1), Slug (SNAIL2) and Twist (TWIST1). **(F)** SK-Hep-3b or MHCC97-H cells were transfected with control vector or PYGB knockdown vector. The protein expression of *N*-cadherin, *E*-cadherin, β-catenin, Snail, Slug and Twist were analyzed 48 h later.

Bioinformatics analysis was performed to identify the potential mechanisms of PYGB in HCC using TCGA HCC dataset. Differential expressions of genes in HCC patients with high or low PYGB expression were analyzed by GSVA (Figure 5A). Gene function was also analyzed by GO and KEGG pathway enrichment analyses (Figures 5B,C). GSEA was performed and the results indicated that high PYGB expression was positively correlated with invasiveness-associated gene signature and epithelial to mesenchymal transition (EMT)-associated gene signature (Figure 5D). Moreover, the relationships between PYGB and the genes in these pathways were analyzed. PYGB expression was positive correlated with the mRNA expressions of *E*-cadherin (CDH1), *N*-cadherin (CDH2), β-catenin (CTNNB1), Snail (SNAIL1), Slug (SNAIL2) and Twist (TWIST1) (Figure 5E). To further validate the molecular mechanisms of PYGB regulation in HCC on the signal pathways of invasiveness and epithelial to mesenchymal transition, we examined the expression levels of these genes in HCC cells transfected with sh-PYGB. PYGB silencing inhibited the protein expression of *N*-cadherin, β-catenin, Snail, Slug and Twist, except *E*-cadherin (Figure 5F). These results suggest that PYGB might regulate HCC progression by controlling the invasiveness and epithelial to mesenchymal transition.

### PYGB Was Targeted and Regulated by miR-101-3p

**FIGURE 6:**
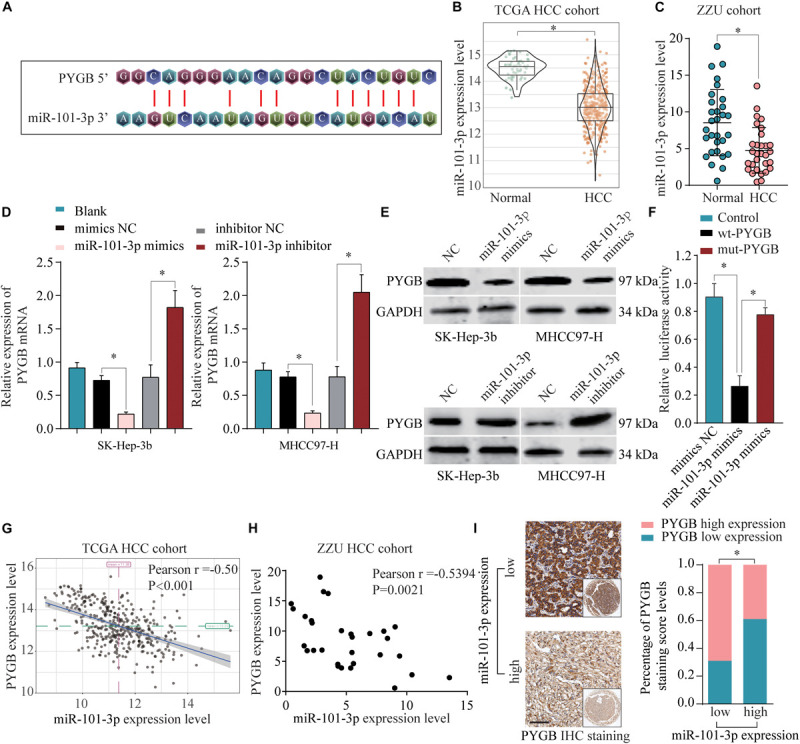
miR-101-3p negatively regulates PYGB expression by binding to its 3′-UTR. **(A)** Complementary binding sequences between miR-101-3p and wild-type or mutated 3′-UTR of PYGB, as predicted by online bioinformatics database TargetScan. **(B,C)** The expression levels of miR-101-3p in HCC tissues and normal liver tissues were analyzed using TCGA HCC cohort and ZZU HCC cohort. **(D,E)** SK-Hep-3b or MHCC97-H cells were transfected with negative control (NC), miR-101-3p mimics, or miR-101-3p inhibitor. The mRNA and protein expression of PYGB were determined by qPCR and western blot 48 h later. **(F)** Luciferase reporter assay was conducted in SK-Hep-3b cells transfected with luciferase reporter vector containing wt-PYGB or mut-PYGB, together with miR-101-3p mimics or negative control. The relative luciferase activity was analyzed 48 h later. **(G,H)** The correlation between PYGB expression and miR-101-3p expression was analyzed using TCGA HCC cohort and ZZU HCC cohort. **(I)** High PYGB expressions were observed in HCC patients with low miR-101-3p expression. *p < 0.05.

To identify the regulatory mechanisms of PYGB in HCC, online bioinformatics database TargetScan was used to predict the potential miRNAs targeting PYGB mRNA. miR-101-3p was predicted to have the putative binding to the 3′-UTR of PYGB mRNA, as shown in Figure 6A. The expression levels of miR-101-3p were analyzed in TCGA HCC cohort and ZZU HCC cohort. Both datasets found lower miR-101-3p expression in HCC tissues compared with that in normal liver tissues (Figures 6B,C), consistent with the previous studies (Su et al., 2009; Shen et al., 2014; Wei et al., 2015; Li C. Y. et al., 2017). Moreover, the mRNA and protein expression of PYGB were down-regulated after miR-101-3p mimics transfection, while up-regulated after inhibition of miR-101-3p (Figures 6D,E). The direct interaction between PYGB 3′-UTR and miR-101-3p was confirmed by luciferase reporter assay. Overexpression of miR-101-3p inhibited the luciferase activity in wt-PYGB transfected cells, but not in mut-PYGB transfected cells (Figure 6F). In addition, Pearson analysis demonstrated a negative correlation between PYGB expression and miR-101-3p expression in TCGA HCC cohort and ZZU HCC cohort (Figures 6G,H). Consistent with these results, high PYGB expressions were observed in HCC patients with low miR-101-3p expression (Figure 6I). These data indicate that miR-101-3p might function as a regulator of PYGB in HCC by directly targeting PYGB.

### miR-101-3p Regulates HCC Cell Proliferation, Invasion and Migration via Targeting PYGB

**FIGURE 7:**
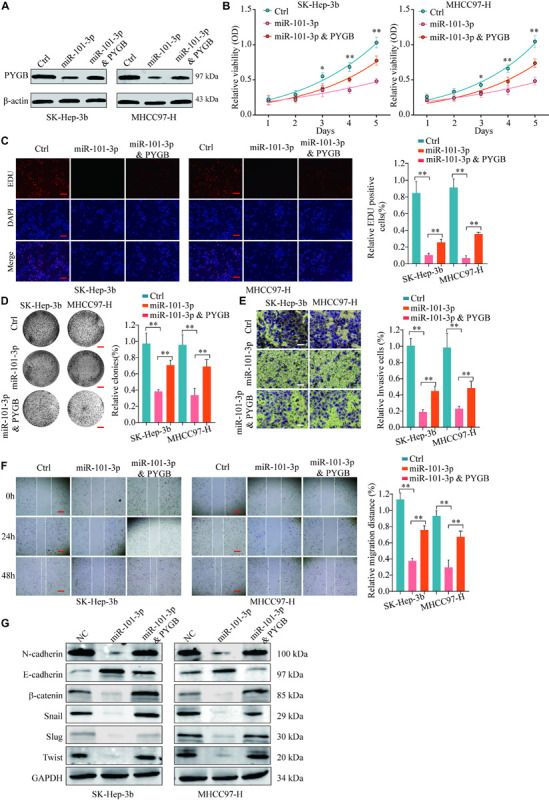
miR-101-3p regulates HCC cell proliferation, invasion and migration via targeting PYGB. SK-Hep-3b or MHCC97-H cells were transfected with negative control, miR-101-3p mimics or miR-101-3p mimics and PYGB overexpression vector. **(A)** The protein expression of PYGB was examined by western blot. **(B–D)** Cell viability, DNA synthesis and colony formation of HCC cells were evaluated using CCK-8 assay **(B)**, EdU assay **(C)**, and colony formation assay **(D)**. **(E,F)** Cell invasion and migration of HCC cells were evaluated using transwell assay and wound healing assay. **(G)** The protein expression of *N*-cadherin, *E*-cadherin, β-catenin, Snail, Slug and Twist in HCC cells were examined by western blot **p < 0.01.

To further validate the regulatory mechanisms of miR-101-3p/PYGB in HCC, HCC cells were transfected with miR-101-3p mimics and/or pcDNA3.1-PYGB. The expressions of PYGB were downregulated after miR-101-3p mimics transfection, but restored by pcDNA3.1-PYGB administration (Figure 7A). Functional assays showed that cell viability, DNA synthesis and colony formation ability of HCC cells were inhibited by miR-101-3p overexpression using CCK-8 assay, EdU assay and colony formation assay, while the inhibition were dampened by PYGB overexpression (Figures 7B–D). PYGB overexpression also rescued the cell invasion and migration ability in HCC cells transfected with miR-101-3p mimics, as demonstrated by transwell assay and wound-healing assay (Figures 7E,F), indicating that PYGB could attenuate the inhibition effect of miR-101-3p overexpression. In addition, the protein expression of *N*-cadherin, β-catenin, Snail, Slug and Twist in HCC cells were inhibited after miR-101-3p mimics transfection, but restored by PYGB overexpression (Figure 7G).

**FIGURE 8:**
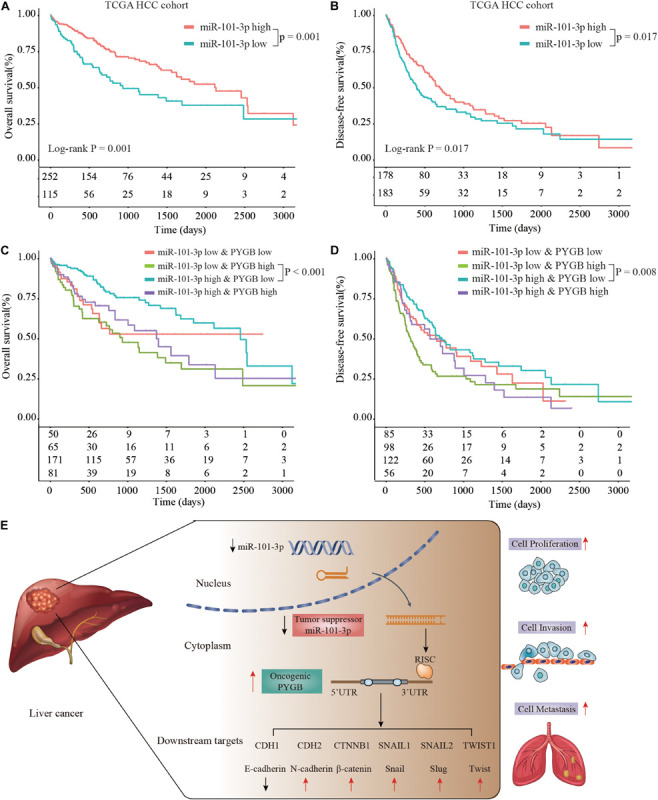
Prognosis analysis of patients with high or low expression of miR-101-3p and PYGB in TCGA HCC cohort. **(A,B)** Kaplan-Meier analysis of the OS and DFS in HCC patients with high or low miR-101-3p expression in TCGA HCC cohort. **(C,D)** Kaplan–Meier analysis of the OS and DFS in HCC patients with different expression levels of miR-101-3p and PYGB. **(E)** Schematic representation of miR-101-3p/PYGB axis promoting HCC tumorigenesis.

Moreover, Kaplan–Meier analysis suggested that patients with low miR-101-3p expression had poor OS and DFS compared with that in patients with high miR-101-3p expression in TCGA HCC cohort (Figures 8A,B). More interestingly, patients with high PYGB expression and low miR-101-3p expression possessed worse OS and DFS than that in patients with low PYGB expression and high miR-101-3p expression (Figures 8C,D). These results confirm that miR-101-3p/PYGB axis has an important role in HCC tumorigenesis (Figure 8E).

## Discussion

PYGB is an enzyme catalyzing the rate-determining step from glycogen to glucose 1-phosphate, and involved in carbohydrate metabolism (Gelinas et al., 1989; Pudil et al., 2010). In cancers, PYGB attracts observation for its nuclear localization in some gastrointestinal carcinoma (Uno et al., 1998). Dysregulated expression of PYGB in a series of cancers was discovered in last two decades (Uno et al., 1998; Tashima et al., 2000; Lee et al., 2006; Wang et al., 2018). In HCC, mouse monoclonal anti-HCA antibody staining indicated PYGB could be served as a potential biomarker for HCC diagnosis (Zhou et al., 2013), which showed the high expression of PYGB in HCC. In this study, we focused on the function and regulatory mechanism of PYGB in HCC development and metastasis.

Previous studies have indicated that PYGB were dysregulated and might be served as a metabolic target for clinical application. For instance, upregulation of PYGB was reported in several cancers, such as colorectal cancer (Tashima et al., 2000), prostate cancer (Wang et al., 2018) NSCLC (Lee et al., 2006) and ovarian cancer (Zhou et al., 2019). High PYGB expression was positively correlated with poor prognosis of ovarian cancer patients (Zhou et al., 2019). Here, we found that mRNA expression levels of PYGB were enhanced in tumor tissues such as HCC, prostate adenocarcinoma (PRAD), colon cancer (COAD), lung cancer (LUAD) and lung squamous cell carcinoma (LUSC), consistent with the results reported by previous studies (Uno et al., 1998; Tashima et al., 2000; Lee et al., 2006; Zhou et al., 2013; Wang et al., 2018). Moreover, we further confirmed the high mRNA and protein expression of PYGB using GEO datasets and ZZU HCC cohort, respectively. Importantly, we found that patients with high PYGB expression had poorer OS than patients with low PYGB expression. PYGB expression, as well as TNM stages and vascular invasion, was an independent risk factor for HCC prognosis. These results indicated that PYGB was highly expressed and could be served as a prognostic biomarker for HCC patients.

Previous study has indicated that glucose utilization via glycogen phosphorylase sustains proliferation and prevents premature senescence in cancer cells (Favaro et al., 2012). Emerging evidence has hinted that PYGB, the number of glycogen phosphorylase family, participates in tumor progression. PYGB knockdown inhibited cell proliferation in human osteosarcoma cell lines (Zhang et al., 2018), and suppressed cell growth and promotes the apoptosis of prostate cancer cells (Wang et al., 2018). In ovarian cancer cells, silencing PYGB inhibited cell proliferation, invasion and migration (Zhou et al., 2019). Inhibition of PYGB expression resulted in decreased glycogen utilization, wound-healing capability, and invasive potential of breast cancer cells (Altemus et al., 2019). To characterize the function of PYGB in HCC, *in vitro* experiments were performed. We found that PYGB knockdown suppressed cell growth and proliferation, colony formation, invasion and migration of HCC cells. Furthermore, silencing PYGB inhibited the tumor growth and metastasis *in vivo*. These data suggest that PYGB play a critical role in HCC growth and metastasis, and might be served as a therapeutic target for inhibiting HCC growth and metastasis.

We further investigated the molecular mechanisms of PYGB in HCC progression. GSEA analysis indicated that PYGB expression was positively correlated with survival down-associated gene signature, invasiveness-associated gene signature and EMT-associated gene signature. Studies have shown that EMT played a critical role in the cell growth, invasion and metastasis of cancer cells (Salt et al., 2014; Nieto et al., 2016; Shen et al., 2019), which were consistent with the roles of PYGB in HCC growth and metastasis. We found that PYGB expression was positively associated with the mRNA expression of EMT-related genes including *N*-cadherin, β-catenin, Snail, Slug and Twist. Moreover, protein expression levels of *N*-cadherin, β-catenin, Snail, Slug and Twist were downregulated in HCC cells after PYGB knockdown. These results indicated that PYGB might be involved in HCC progression by enhancing the invasiveness and EMT process of HCC cells.

Referring to the regulatory mechanisms of PYGB in HCC, bioinformatics analysis indicated that miR-101-3p was a potential candidate for targeting PYGB mRNA. Mounting evidences proved that miR-101 was downregulated in HCC and the low expression of miR-101 was associated with poor prognosis of HCC patients (Su et al., 2009; Shen et al., 2014; Wei et al., 2015; Li C. Y. et al., 2017; Wakasugi et al., 2018; Weidle et al., 2020). More importantly, recent studies have reported that miR-101 suppressed proliferation and migration of HCC cells by targeting the HGF/c-Met pathway (Liu et al., 2019), PI3K/Akt/mTOR pathway (Zhang et al., 2019) and so on. In addition, miR-101 played an vital role in EZH2-induced sorafenib resistance (Hu et al., 2019) and cancer-associated fibroblasts (Yang et al., 2016) in hepatocellular carcinoma. In this study, we found that there was a negative correlation between PYGB expression and miR-101-3p expression in TCGA HCC cohort and ZZU HCC cohort. Patients with high PYGB expression and low miR-101-3p expression possessed poorer OS and DFS that patient with low PYGB expression and high miR-101-3p expression. Moreover, the directly inhibition of PYGB expression by miR-101-3p overexpression was verified *in vitro*. We also found that cell proliferation, invasion and migration of HCC cells were inhibited by miR-101-3p, while the inhibition could be dampened by PYGB overexpression. Meanwhile, PYGB overexpression could restore the expression of *N*-cadherin, β-catenin, Snail, Slug and Twist inhibited by miR-101-3p in HCC cells. These results indicated that miR-101-3p might regulate HCC tumorigenesis and metastasis via targeting PYGB.

Based on our findings, PYGB may be served as therapeutic target for HCC patients, which provides a novel therapeutic strategy targeting PYGB by miR-101-3p. We expect that miR-101-3p could be optimized in HCC tissue specificity by some technology, such as nanotechnology in the future, which is beneficial for the precise targeted treatment of HCC patients.

## Conclusion

Collectively, we find that PYGB is highly expressed in HCC tissues and PYGB overexpression is associated with a poor prognosis of HCC patients. PYGB promotes HCC cell tumorigenesis and metastatic progression. Moreover, PYGB is identified as a direct target of miR-101-3p and miR-101-3p might regulate HCC development via targeting PYGB. In short, our results suggest that PYGB may be served as a novel prognostic biomarker and therapeutic target for improving the prognosis of HCC patients.

## Data Availability Statement

All datasets generated for this study are included in the article/supplementary material.

## Ethics Statement

Written informed consent was obtained from each patient. The study was performed in accordance with the Helsinki Declaration and Rules of Good Clinical Practice and approval by the First Affiliated Hospital of Zhengzhou University.

## Author Contributions

GC and SS designed the study. GC, HFW, WL, JX, WS, ZZ, XW, HL, and XL collected the clinical data and performed the experiments. HFW, WL, JX, WS, and LL analyzed the data and established the model. GC, HFW, and SS wrote the manuscript. All the authors reviewed and approved the manuscript.

## Conflict of Interest

The authors declare that the research was conducted in the absence of any commercial or financial relationships that could be construed as a potential conflict of interest.

## Footnotes

1 http://www.ncbi.nlm.nih.gov/geo/

2 https://tcga-data.nci.nih.gov/tcga/
